# Free-ranging bats combine three different cognitive processes for roost localization

**DOI:** 10.1007/s00442-020-04634-8

**Published:** 2020-03-31

**Authors:** Jesús R. Hernández-Montero, Christine Reusch, Ralph Simon, Caroline Regina Schöner, Gerald Kerth

**Affiliations:** 1 Zoological Institute and Museum, Applied Zoology and Nature Conservation, Greifswald University, Loitzerstraße 26, 17489 Greifswald, Germany; 2 Department of Ecological Science/Animal Ecology, Faculty of Earth and Life Sciences, Vrije Universiteit Amsterdam, Amsterdam, The Netherlands

**Keywords:** Cognition, Associative learning, Spatial memory, Social information, *Myotis bechsteinii*

## Abstract

Animals have evolved different cognitive processes to localize crucial resources that are difficult to find. Relevant cognitive processes such as associative learning and spatial memory have commonly been studied in a foraging related context under controlled laboratory conditions. However, in natural environments, animals can use multiple cognitive processes to localize resources. In this field study, we used a pairwise choice experiment and automatic roost monitoring to assess how individually marked, free-ranging Bechstein’s bats belonging to two different colonies use associative learning, spatial memory and social information when localizing suitable day roosts. To our knowledge, this study tests for the first time how associative learning, spatial memory and social information are used in the process of roost localization in bats under the natural conditions. We show that, when searching for new roosts, bats used associative learning to discriminate between suitable and unsuitable roosts. For re-localizing previously occupied roosts, bats used spatial memory rather than associative learning. Moreover, bats significantly improved the localization of suitable unfamiliar roosts and tended to increase their accuracy to re-localize previously occupied day roosts using social information. Our field experiments suggest that Bechstein’s bats make hierarchical use of different cognitive processes when localizing day roosts. More generally, our study underlines that evaluating different cues under natural conditions is fundamental to understanding how natural selection has shaped the cognitive processes used for localizing resources.

**Electronic supplementary material:**

The online version of this article (10.1007/s00442-020-04634-8) contains supplementary material, which is available to authorized users.

## Introduction

Localizing resources that are crucial for survival and reproduction such as food or shelters can be time and energy consuming (Dall and Johnstone 2002). Consequently, animals have evolved sensory systems and cognitive processes that maximize net benefits by minimizing resource location costs (Niven and Laughlin 2008). Cognitive processes allow animals to improve their localization of resources through experience and interaction with environmental cues (Brown 2012) to which animals need to respond according to their relevance (Fawcett and Johnstone 2003; Shettleworth 2005). Cues can be object-specific (i.e., colors, shapes), spatial (position of objects in space) (Herborn et al. 2010) and social (when derived from the activity of other animals; Zentall 2006). These cue types can be related to different cognitive processes, among them: (1) associative learning, (2) spatial memory and (3) social information use.

Associative learning is a cognitive process that allows animals to build neural representations of associations between cues and resources (Dukas 1999). This cognitive process has been studied in a wide range of organisms and behavioral contexts such as foraging, predator avoidance, prey recognition, mate choice, navigation and shelter selection (Morand-Ferron 2017). The generalization of the learned cue-resource associations increases the efficiency of animals to locate resources and to assess their quality.

Spatial memory is another cognitive process for memorizing resources’ location after they had been previously discovered (Benhamou 1994). Spatial memory has been intensively studied in relation to foraging ecology, where it differs strongly between food-storing and non-storing species. For example, food-storing bird species rely preferentially on spatial cues over object-specific cues for returning to food locations, while non-storing species appear to use both kinds of cues equally (Brodbeck 1994; McGregor and Healy 1999). Spatial memory is typically favored when resources are spatiotemporally predictable. For example, nectar-feeding animals (e.g., Hymenoptera) depend on their spatial memory to track the distribution of foraging patches and locations already exploited (Orth and Waddington 1997; Chittka et al. 1999).

Finally, social information use is a cognitive process based on associative mechanisms (Heyes 2012) between the activity of individuals (typically a conspecific) that face a similar situation (e.g., foraging, shelter selection) and the outcome of their behavior (Seppänen et al. 2007). Social information use is based on social cues that can be obtained from direct interaction with conspecifics or indirectly from the products of their behavior, such as feces or inadvertently emitted sounds (Danchin et al. 2004; Galef and Laland 2005). Using social information can reduce the costs of localizing and assessing resources that occur if animals use an individual trial–error strategy (Galef and Giraldeau 2001; Thornton and McAuliffe 2006). Thus, when analyzing how animals localize resources, we have to consider the use of social and non-social cues and how such cues affect their performance differently (Jones et al. 2013).

The above mentioned cognitive processes have been commonly studied under laboratory conditions that minimize the variation of the learning context (Morand-Ferron et al. 2016). Laboratory studies have yielded valuable insights into the cognitive abilities of animals. However, experimental conditions rarely match the physical and social environment in which organisms have evolved (Fawcett et al. 2014; Morand-Ferron et al. 2016). Field studies offer the complex environment of the species; but confounding factors are often more challenging to control. Cognitive experiments conducted in the wild rely on protocols where animals freely interact with the experimental setup (Thornton and Samson 2012; Morand-Ferron et al. 2016). This voluntary participation, coupled with automatic data collection devices, can reveal the behavioral performance of the target species (Morand-Ferron et al. 2015; Cauchoix et al. 2017).

Resource localization has been studied primarily in relation to foraging behavior, but it can also be observed in other contexts. The roost searching behavior of forest-dwelling bats provides an interesting model to explore how different cognitive processes may be involved in localizing resources. Finding suitable roosts can be time and energy consuming given the typically low availability of roosts and the limitations of sensory ranges (e.g., vision, echolocation) for detecting them. Hence, bats have to implement searching strategies to decrease the costs of roost finding. A simulation model exploring how bats locate suitable roosts under different scenarios of roost density and perceptual ranges showed that bats can use associative learning, spatial memory and social information to compensate for their sensory limitations (Ruczyński and Bartoń 2012). Empirical studies suggest that bats may search for characteristic elements of roosts to find them (e.g., visual cues, echo roughness, temperature) pointing to their capacity of associative learning (Ruczyński et al. 2007, 2009, 2011). However, associative learning in bats has only been tested in foraging related contexts (Siemers 2001; Simon et al. 2006). Spatial memory has been evidenced in roost localization based on long-term re-use of tree cavities by European forest bats (Lučan et al. 2009). Finally, empirical studies have demonstrated that the use of social information (e.g., eavesdropping on conspecific calls, presence of conspecifics) facilitates roost localization (Kerth and Reckardt 2003; Ruczyński et al. 2009; Sagot et al. 2018).

With a field experiment, we aimed to disentangle the interplay of associative learning, spatial memory and social information use in the localization of day roosts in two free-ranging colonies of RFID-tagged Bechstein’s bats (*Myotis bechsteinii*). For this purpose, we used a pairwise discrimination task protocol. We introduced experimental pairs of bat boxes composed of an unsuitable and a suitable roost, hanging side by side at the same tree. We marked each box type (suitable vs. unsuitable) with a distinctive echo-reflective cue, which allowed for an association between this cue and the suitability of the respective box-type for roosting. The use of spatial memory was assessed by swapping the position of the experimental boxes within the experimental pair after the suitable box of the respective pair has ben used as a day roost. Finally, social information use was determined depending on whether the bats visited the boxes in groups or individually. We continuously monitored all experimental boxes for the presence of individually marked bats using automatic RFID-loggers.

We expected that the bats would learn to associate the echo-acoustic cue of the boxes with their suitability as a day roost. If associative learning takes place, we predicted (i) that as bats gain experience with both experimental box types, they would discover a higher number of experimental pairs (pair discovery) by first visiting the suitable box. Among previously discovered roosts, we experimentally assessed how bats re-localize suitable boxes: either via cue-directed search (associative learning of the echo-reflective cue) or using spatial memory (location of the box at a tree). Given the predictable location of previously discovered natural roosts (e.g., tree cavities), (ii) we predicted that bats should rely on spatial memory for re-localizing suitable boxes that they had used as day roost before. Finally, given previous evidence of information transfer about roosts in Bechstein’s bats (Kerth and Reckardt 2003), we predicted that (iii) colony members rely strongly on social information for (re)localizing suitable roosts. By investigating the interaction of multiple cognitive abilities, this study gives insights into how free-ranging animals use different cues available in their environment and thus whether individuals favor one cognitive process over another (Fawcett and Johnstone 2003).

## Materials and methods

### Study species, field sites and monitoring

In forest-dwelling Bechstein’s bat, females are highly philopatric to their natal colony where they communally breed during summer. Bechstein’s bat colonies show a fission–fusion behavior and colony members switch day roosts (tree cavities and bat boxes) on a regular basis (Kerth et al. 2011). As a consequence, a colony may use up to 50 different roosts during one breeding season. Previous studies have shown that individuals regularly explore new potential roosts by visiting them over several nights before using them as a day roost (Kerth and Reckardt 2003; Fleischmann and Kerth 2014).

We conducted our study during two breeding seasons (May to September) in the home range of two Bechstein’s bat colonies (Colony ‘BS’ in 2016 and ‘UA’ in 2018) living in deciduous forests close to the city of Würzburg, Germany. Both colonies regularly roost in bat boxes (Schwegler model 2FN) and have been monitored for over 20 years (Kerth et al. 2002). During our study, the colonies comprised 12 (BS in 2016) and 22 (UA in 2018) adult females previously marked with RFID-tags (Trovan, Germany). Between May and September, we monitored all previously installed boxes (112 in BS; 49 in UA) and newly installed experimental boxes on a daily basis (see next section). We installed automatic RFID-tag loggers (LID 650; EURO ID, Germany), powered by a battery (12 V/3.6 Ah) with the detection antenna (6 cm × 4 cm) placed in the outer entrance of the boxes. We programmed loggers to record individual ID, date and time for each bat passing through the antenna between 19:00 and 07:00. We placed loggers at all experimental boxes at the beginning of the experiment, and additionally at all boxes currently in use as day roosts based on our daily roost monitoring (Kerth and Reckardt 2003). During daily roost monitoring, we checked all bat boxes in the area for roosting bats, which can be seen with a flashlight through the entrance of the boxes (Kerth and König 1999). This automatic monitoring method gives continuous monitoring data recording about 97% of the bats present in the boxes (Kerth and Reckardt 2003).

### Experimental setup

To investigate the cognitive skills of bats for localizing new roosts, we placed ten experimental pairs of bat boxes within the home ranges of both colonies. An experimental pair consisted of a ‘suitable’ and an ‘unsuitable’ box placed side by side on the same tree separated from each other by 25 cm (Fig. S1.1). These bat boxes have two entrances: an interior and exterior. Suitable boxes had both entrances open, allowing the bats to roost inside. Unsuitable boxes had the interior entrance blocked with a plastic mesh allowing the bats to explore entrance area of the box but preventing them from using it as a day roosts (see Kerth and Reckardt 2003 for details). We randomize the position of each box (left or right) at a given tree. We removed and cleaned all the experimental boxes in September when bats began to leave the study area for hibernation, to prevent any parasite or odor cues from carrying over between years.

Each box type was associated with a distinctive echo-reflective cue. As echo-reflective cues, we used hollow concave hemispheres made of plexiglass with a radius of 40 and 50 mm. We glued the hemispheres to the lid of the boxes. We marked each suitable box with a smaller hemisphere (40 mm) and each unsuitable box with a larger hemisphere (50 mm). We used hollow hemispheres as echo-reflective cues because they reflect an echo with a broad directivity and a distinct spectral signature. Previous research has shown that bats are able to easily recognize such hemispheres in clutter rich surrounding, and distinguish even small differences in size (Simon et al. 2006).

To ensure that each hemisphere size had a distinctive echo-signature, we characterized their spatio-spectral features using a biomimetic sonar head with a 1/4” free-field microphone (G.R.A.S. Sound & Vibration, Denmark) and a custom-built double-layer electro-mechanical film (EMFI) speaker (Simon et al. 2006). We ensonified the hemispheres at a distance of 40 cm, with artificial chirp signals (40–160 kHz, 3 ms) comprising the call frequency range of Bechstein’s bats (range 42–112 kHz, frequency with the most energy: 73 kHz; Parsons and Jones 2000). We measured the spectral target strength (dB) around the hemispheres’ concave side. We defined 0º as the opening plane of the hemisphere being perpendicular to the sound propagation and measured along the azimuth plane from ± 90º in increments of 1.8º (see spectral directional plot; Fig. S1.2).

### Data processing

We assessed the cognitive skills involved in roost localization by analyzing the nightly roost visitation pattern of each bat obtained from the logger data. We considered only the first record per bat to a given box per night as a ‘visit’ to avoid pseudo-replication due to revisits to the same box during the night. We chronologically arranged the visits of each bat to the experimental boxes so that we had a record of every consecutive visit according to the box type visited.

We recorded the following variables for each visit: (i) box type, ‘suitable’ or ‘unsuitable’; (ii) previous experience of a bat, defined as either ‘naïve’ or ‘experienced’, depending on whether or not the bat had visited a given box for the first time; (iii) information type, categorized as ‘non-social’ or ‘social’. We considered visits as ‘non-social’ when no other colony members had been recorded within 1 min, and ‘social’ information as visits involving more than one bat (a naïve bat arriving within 1 min of an experienced bat; see Supplementary Material S2). We defined the visit of the first bat of an all-naïve group as ‘non-social’ and the other visiting bats as ‘social’ because those latter bats could have observed the behavior of the first bat. To inspect the consistency of our results, we performed statistical analyses using alternative time spans (30 and 180 s; see Supplementary Material S3). We defined (iv) ‘pair discovery’ as the first visit of each bat to an experimental pair. According to the first box type visited, a ‘pair discovery’ can be ‘suitable’ or ‘unsuitable.’ Finally, we recorded (v) the cumulative number of previous ‘suitable’ and ‘unsuitable’ visits for every ‘pair discovery’ event to quantify the previous experience of an individual bat with the two different box types at the moment of discovering a new experimental pair.

### Data analysis

To assess associative learning, spatial memory and social information use, we pooled the data of the two colonies from the years 2016 (BS) and 2018 (UA) because both colonies behaved similarly concerning to pair discoveries (see Supplementary Material S4). Before assessing for associative learning, we tested whether bats had an initial preference for a particular box type (i.e., its echo-acoustic signature) using a binomial test with an expected proportion of 0.50. We computed the binomial test only with those individuals using non-social information in their very first visit to an experimental box to rule out that the bats merely copied the behavior of conspecifics. We performed all statistical analyses in R v.3.4.3 (R Core Team 2017).

#### Associative learning

To assess for associative learning of the echo-reflective cue, we compared the number of pair discoveries recorded for each box type (suitable vs. unsuitable) when bats used non-social information with a Wilcoxon matched-pair signed-rank tests (paired per individual bat). Moreover, we carried out generalized additive mixed models (GAMM) to examine the likelihood that an individual bat discovers a suitable box as a function of its previous experience at the time of discovery and the information type used. We used GAMM because we expected a non-linear relationship between the probability of discovering a suitable box and the numeric covariates (Zuur et al. 2009). We set the box type visited (suitable: 1, unsuitable: 0) as the response variable. We included the following fixed effects: the cumulative number of visits to suitable and unsuitable boxes, and the information type used (non-social or social). We included random effects for individual identity and colony to control for possible dependence due to repeated measures or colony effects. Because the response variable was binary, we used a binomial model and a logit link function. To select the best minimal model, we built alternative models using an all-subset approach and ranked them according to their Akaike Information Criterion corrected for small sample sizes (AICc; Supplementary Table S5.1). We carried out all GAMM models using the mgcv R-package. Fit quality assessment of the minimal model is available in the Supplementary Material S5.

#### Spatial memory

To test whether bats use spatial memory to re-localize previously occupied roosts, we swapped boxes’ positions within the same experimental pair (left or right on the same tree) after the bats had left the suitable box used as a day roost for at least 1 day. We cleaned the boxes with clear water before swapping them to avoid the potential use of olfactory cues. Association contingencies remained the same before and after swapping the position of the boxes.

We recorded the box type that each bat visited using non-social information before and after the swap as follows. Before swapping boxes within a pair, we recorded the last box type that each bat visited, from the given pair, before starting to use the suitable box as a day roost. After swapping the boxes, we recorded the box type of the first revisit to the respective experimental pair of each bat. To examine the effect of swapping the box in the re-localization of the suitable box, we calculated the mean proportion of bats visiting the suitable box before and after the swap using non-social information (henceforth $\overline{{\hat{p}_{{{\text{before}} - {\text{ns}}}} }}$ and $\overline{{\hat{p}_{{{\text{after}} - {\text{ns}}}} }}$, respectively). Because bats often visit a suitable box several times before using it as a day roosts (Kerth et al. 2006), we expected $\overline{{\hat{p}_{{{\text{before}} - {\text{ns}}}} }}$ to be close to one, i.e., almost all visits directly prior to swapping were to suitable boxes. If bats use spatial memory for re-localizing the suitable box, we expected $\overline{{\hat{p}_{{{\text{after}} - {\text{ns}}}} }}$ to be close to zero i.e., the unsuitable box, now at the position where the suitable box was previously located, would be visited first. On the contrary, if bats employed a cue-directed search (associative learning; i.e., the echo-reflective hemispheres) to re-localize the suitable box, we expected $\overline{{\hat{p}_{{{\text{after}} - {\text{ns}}}} }}$ to remain close to one after the swap.

Because only seven of 20 experimental pairs were revisited after the swap, we used a permutation test to assess whether our observed $\overline{{\hat{p}_{{{\text{before}} - {\text{ns}}}} }}$ and $\overline{{\hat{p}_{{{\text{after}} - {\text{ns}}}} }}$ deviated from a random distribution. For each mean proportion (before and after), we generated a permutated data set (1000 permutations) by sampling the observed visits with replacement and randomly allocating them in one of the two box types (suitable or unsuitable). Then, we calculated the mean proportion (*n* = 7 for each permutation) by dividing the total number of visits to suitable boxes by the total number of visits (Crawley 2013). We compute the probability of getting our observed $\overline{{\hat{p}_{{{\text{before}} - {\text{ns}}}} }}$ and $\overline{{\hat{p}_{{{\text{after}} - {\text{ns}}}} }}$ from the permutated data sets with normal distribution using their associated *Z* score. Observed values deviate from random when they have a probability lower than 0.05. To assess whether swapping boxes within a pair (before vs. after) had a significant effect in the proportion of bats visiting the suitable box, we conducted a generalized linear mixed model (GLMM) with binomial distribution fitting the experimental pair identity as a random effect.

#### Social information use

In the previously described analyses, we assessed associative learning (pair discovery) and spatial memory only when bats used non-social information. However, if social information use improves the roosts localization, we would expect a higher number of pair discoveries using social information (i.e., arriving with other colony members) than when bats use non-social information (i.e., arriving alone). Thus, for each box type (suitable and unsuitable), we compared the number of pair discoveries between information types (non-social vs. social) using the Wilcoxon matched-pair signed-rank test. We paired the data from each information type per individual bat.

We also assessed whether social information use improves the re-localization of previously occupied roosts. For bats using social information, we calculated the mean proportion of bats visiting suitable roosts before and after swapping the position of the box sides using social information (henceforth $\overline{{\hat{p}_{{{\text{before}} - {\text{s}}}} }}$ and $\overline{{\hat{p}_{{{\text{after}} - {\text{s}}}} }}$, respectively). We assessed whether the observed $\overline{{\hat{p}_{{{\text{before}} - {\text{s}}}} }}$ and $\overline{{\hat{p}_{{{\text{after}} - {\text{s}}}} }}$ deviates from a random distribution using a permutation test as described above. We evaluated the effect of swapping boxes within a pair in the mean proportion of bats visiting the suitable box using social information with a binomial GLMM. We specified the experimental pair a random effect.

Finally, we expected that bats were more successful in re-localizing suitable roosts when using social information than when they use non-social information ($\overline{{\hat{p}_{{{\text{after}} - {\text{s}}}} }}$ > $\overline{{\hat{p}_{{{\text{after}} - {\text{ns}}}} }}$). We assessed the effect of the information type for re-localizing the suitable box using a binomial GLMM. We fitted the information type (non-social vs. social) as the explanatory factor and the experimental pair as a random effect.

## Results

We collected data from 12 bats in 2016 and 22 bats in 2018 for a total of 34 adult female bats. Five out of nine individuals using non-social information in their first visit of an experimental box visited the suitable box (binomial test, *P* = 0.55). This result suggests that the bats could not detect whether the boxes were suitable or unsuitable before visiting at least one of the two box types (compare Kerth and Reckardt 2003).

### Associative learning

**Fig. 1:**
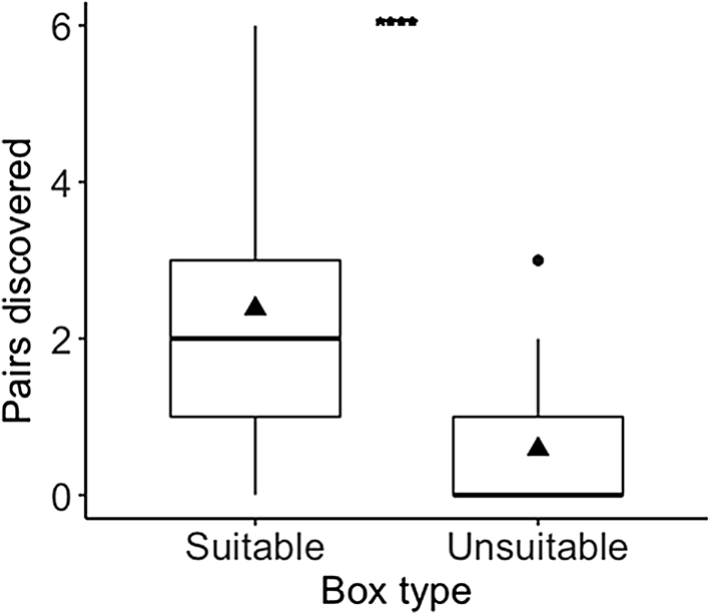
Boxplot of the number of pairs discovered per box type performed by female Bechstein’s bats (*n* = 34) using non-social information (individuals arriving alone). Triangles represent the mean. Results from Wilcoxon matched-pair signed-rank test displayed as ****P ≤ 0.0001

**Fig. 2:**
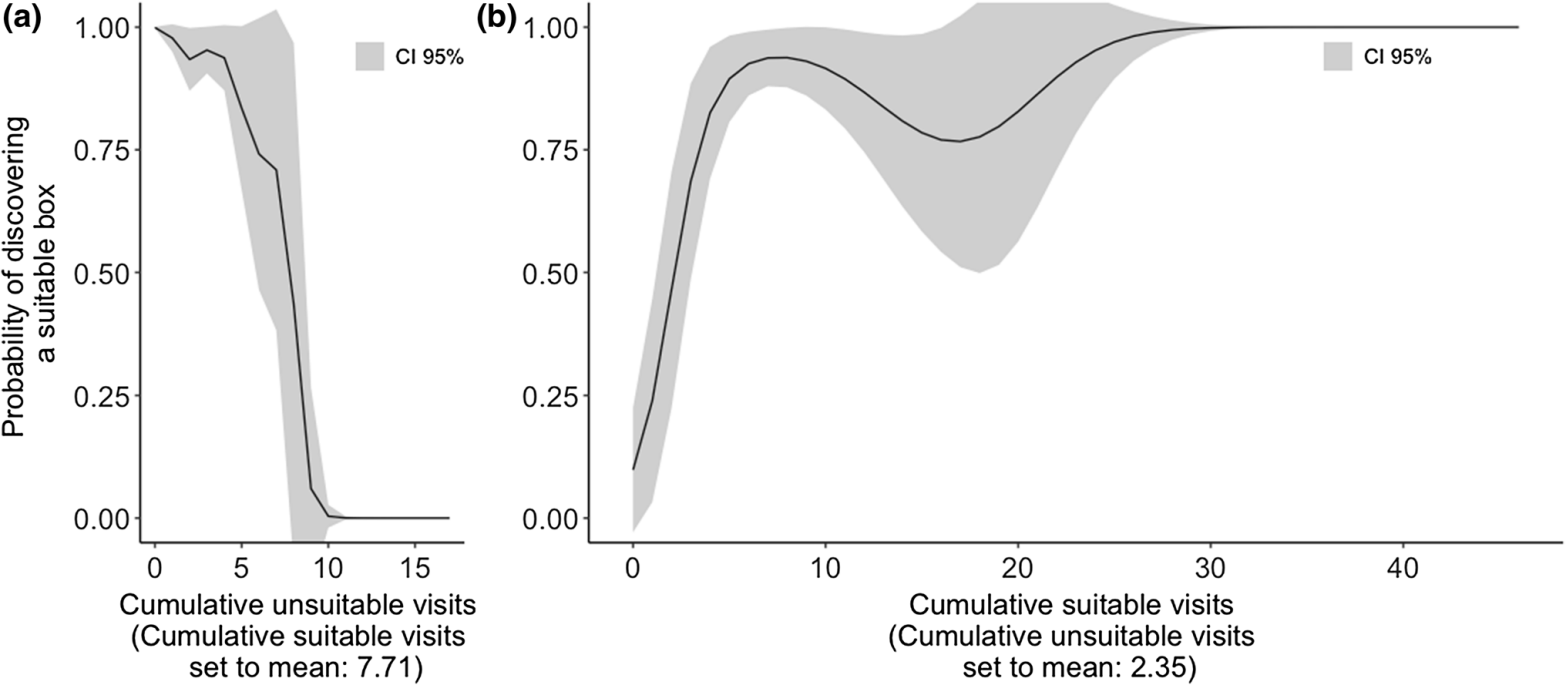
Predicted probability to discover box pairs by visiting suitable roosts depending on the cumulative number of visits to unsuitable (**a**) and suitable boxes (**b**) by female Bechstein’s bats (*n* = 34). The estimate and its 95% confidence interval were based on our best fitted generalized additive mixed model (GAMM) including the cumulative number of suitable and unsuitable visits as fixed effects; bat identity and colony were random intercepts. While investigating the effect of each factor separately the remaining factor was set to its mean. Given the similar behavior of the two colonies, we present only the estimated probabilities for the Blutsee colony as a representative

Bats using non-social information discovered more experimental pairs by first visiting the suitable box (Wilcoxon matched-pair signed-rank: *Z* = 4.553, *P* < 0.0001, *n* = 34 bats; Fig. 1). According to our top-ranked model, which explained 40% of the deviance (Table S5.1, Fig. 2), the probability of discovering a suitable box was best explained by the additive effect of the cumulative number of visits to suitable and unsuitable boxes performed by each individual (see Table 1). Neither random effect (individual identity and colony) significantly affected an individual’s probability to first visit the suitable box (Table 1).

**Table 1:** Generalized additive mixed model on factors affecting the probability that Bechstein’s bats discovering a suitable box

Parametric coefficient	Estimate	SE	*Z*	*P* value
(Intercept)	2.467	0.334	7.382	< 1.56e^−12^

Smooth terms		*edf*	*χ*^2^	*P* value
Cumulative suitable visits		4.792	38.544	< 6.81e^−6^
Cumulative unsuitable visits		6.301	35.233	< 1.25e^−4^
Colony (Random factor)		0.254	0.269	0.301
Bat identity (Random factor)		0.00	0.00	0.712

Model parameter	Deviance explained	*R*^2^	UBRE	Sample size
	40%	0.403	− 0.347	*N*_dicov_ = 270 *N*_bats_ = 34

Data were fitted to a binomial distribution with logit-link function and binary response (0 = unsuitable box, 1 = suitable box). The number of pair discoveries (*N*_discov_) and bats (*N*_bats_) that were included in the model are given

### Spatial memory

The mean proportion of bats visiting the suitable box significantly decreased after swapping the position of the boxes within the experimental pair ($\overline{{\hat{p}_{{{\text{before}} - {\text{ns}}}} }}$: 0.80; $\overline{{\hat{p}_{{{\text{after}} - {\text{ns}}}} }}$: 0.28; GLMM: *df* = 11, *Z* = − 4.44, *P* < 0.0001). Both values deviated from a random distribution ($\overline{{\hat{p}_{{{\text{before}} - {\text{ns}}}} }}$: *Z* score = 2.84, *P* = 0.002; $\overline{{\hat{p}_{{{\text{before}} - {\text{ns}}}} }}$: *Z* score = − 2.70, *P* = 0.003).

### Social information use

**Fig. 3:**
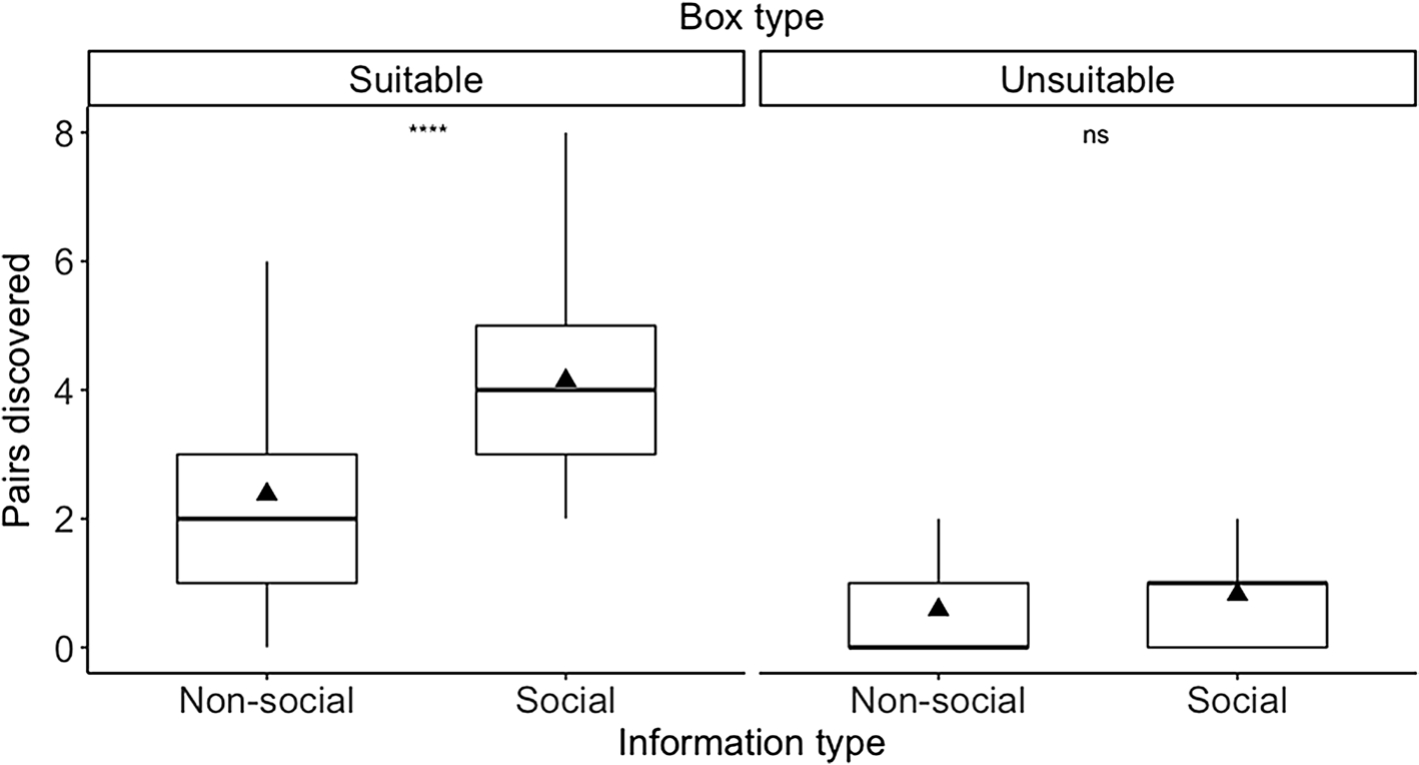
Box plot of the number of pairs discovered performed by female Bechstein’s bats (*n* = 34) using non-social (individuals arriving alone) and social information (individuals arriving within a group of bats) per box type. Triangles represent the mean. Results from Wilcoxon matched-pair signed-rank test displayed as: ns *P* > 0.05; ****P ≤ 0.0001

For suitable boxes, bats performed a higher number of pair discoveries (*P* = 0.001) using social information compared to cases when they used non-social information (Fig. 3; Table 2). For unsuitable boxes, we did not observe a difference between information types (*P* = 0.15; Fig. 3, Table 2).

**Table 2:** Total number of pair discoveries per box type (mean ± SD) and comparisons between information types (Z: Wilcoxon matched-pair signed-rank test), *n* = 34 bats

Box type	Information type		*Z*	*P* value
	Non-social	Social		
Suitable	81 (2.38 ± 1.44)	141 (4.15 ± 1.48)	3.286	0.001
Unsuitable	20 (0.58 ± 0.89)	28 (0.82 ± 0.71)	1.410	0.15

The mean proportion of bats visiting the suitable roost significantly decreased after swapping the position of the boxes within the experimental pair ($\overline{{\hat{p}_{{{\text{before}} - {\text{s}}}} }}$: 0.97; $\overline{{\hat{p}_{{{\text{after}} - {\text{s}}}} }}$: 0.56; GLMM: *df* = 11, *Z* = − 4.51, *P* < 0.0001). However, only the proportion before the swap deviated from random ($\overline{{\hat{p}_{{{\text{after}} - {\text{s}}}} }}$: *Z* score = 3.03, *P* = 0.001; $\overline{{{\text{after}} - {\text{s}}}}$: *Z* score = 0.76, *P* = 0.22). The mean proportion after swapping the box positions was significantly higher when bats used social information compared to when bats used non-social information ($\overline{{\hat{p}_{{{\text{after}} - {\text{s}}}} }}$: 0.56 vs. $\overline{{\hat{p}_{{{\text{after}} - {\text{s}}}} }}$: 0.28; GLMM: *df* = 11, *Z* = 3.130, *P* < 0.001).

## Discussion

Our experimental field study demonstrated that free-ranging female Bechstein’s bats used a suite of different cognitive skills to localize their day roosts. Associative learning of an echo-acoustic cue indicating box quality improved the bats’ ability to find new suitable roosts; however, for re-localizing previously occupied roosts, bats relied mostly on spatial memory. The use of social information significantly increased the performance of bats on their first visit to unfamiliar roosts.

We found no evidence that the bats initially preferred one of the two box types. However, as bats gathered experience with the experimental task, they quickly started to discriminate between box types. The higher number of discoveries of suitable boxes compared to unsuitable boxes showed that the bats associated the boxes’ echo-acoustic cues with their suitability (Fig. 1). The cumulative number of visits to suitable and unsuitable boxes best explained how bats learned to associate the boxes’ echo-acoustic signature with their suitability as a day roost. The cumulative number of visits to suitable and unsuitable boxes can be seen as a proxy of experience of the bats with both box types. This indicates that the process of discrimination learning is typically based on the experience of both failure and success (Spence 1936).

Associative learning has previously been demonstrated in a foraging-related task with nectar-feeding bats using artificial echo-acoustic cues like in our experiment (von Helversen 2004; Simon et al. 2006). Our results showed for the first time that bats can also use associative learning for localizing novel roosts, as previously suggested by Ruczyński et al. (2009). We were confident that bats used associative learning to discriminate between box types as we were able to exclude or control for the effects of other learning methods. First, we controlled for social information by only analyzing pair discoveries of bats that used non-social information, thereby excluding individuals that may have instead used social information. Second, our results on associative learning were consistent using alternative time spans for categorizing social information (Fig. S3.1). Lastly, since pair discovery was defined as only the very first visit to a given pair of boxes, spatial memory could not influence the decision of the bats. Overall, the experiment showed that the bats quickly learned to associate the box’s suitability with its echo-reflective cue. Therefore, by swapping the position of boxes within pairs, we were able to investigate the relative use of associative learning and special memory.

While bats used associative learning to find new suitable roosts, our experimental relocation of boxes within a pair showed that bats strongly relied on spatial memory for re-localizing day-roosts. Most of the bats that revisited the experimental pairs, after swapping the position of the boxes, visited the unsuitable box located where the suitable box previously was. This result shows that the bats largely relied on spatial memory when re-localizing a previously occupied roost. Since both boxes still had the distinctive echo-reflector attached to them, this finding suggests that bats prioritize spatial memory over a cue-directed search (associative learning) when revisiting previously discovered suitable roosts.

When we assessed for differences between non-social and social information, we found that the use of social information (arriving at a box together with other colony members) improved the bats’ performance in the localization of suitable roosts. However, we did not observe such an improvement for unsuitable boxes. As bats profit from the presence of conspecifics for thermoregulation (Pretzlaff et al. 2010), we would not expect that they recruit conspecifics to unsuitable roosts (Kerth and Reckardt 2003). Regarding the re-localization of day roosts, we observed a higher mean proportion of bats visiting the suitable box after swapping boxes’ sides using social information compared to non-social information. This suggests that bats were more accurate in re-localizing the suitable box using social information. However, we do not have conclusive evidence since our permutation test showed that our observed $\overline{{\hat{p}_{{{\text{after}} - {\text{s}}}} }}$ did not differ significantly from random chance.

In addition to observing another bat entering a roost, bats might also use other sources of social information not detectable with our RFID-monitoring, such as odor cues or vocalizations at suitable boxes. In another European bat, *Nyctalus noctula*, odor cues have been demonstrated to play a minimal role as social cues for roost localization (Ruczyński et al. 2009). To nevertheless minimize the possible effect of odor cues, deployed experimental boxes were either brand-new (30 out of 40) or thoroughly cleaned before the experiments. Likewise, boxes were cleaned when their position was swapped, making the use of odor cues over other information sources unlikely. Regarding social acoustic cues, we cannot exclude the possibility that bats were attracted by social calls by bats swarming near the entrance of a box (i.e., dawn swarming; Naďo and Kaňuch 2015), or emitted from inside the suitable boxes (Schöner et al. 2010). However, we considered that the arrival pattern of bats at boxes is a good proxy for inferring social information use, including the potential use of social calls from in or around the boxes. Our analysis using time spans of either 60 or 180 s as the cutoff for social visit delineation led to similar results. However, using a 30-s span resulted in no differences between information types for suitable boxes (see Fig. S3.2). In a previous study assessing information transfer in Bechstein’s bats, Kerth and Reckardt (2003) used a 180 s span between records as individuals do not enter a box immediately upon arriving. Using a time span of 30 s may falsely identify social visits as non-social visits if the experienced bat flies around within the direct vicinity of the box for several minutes before entering as has previously been observed (Schöner et al. 2010; Naďo and Kaňuch 2013). Thus, we considered our 60-s span to be a more accurate estimate of social information availability for roost localization than a 30-s span, while simultaneously limiting the false positives that a longer timespan could introduce.

### Do bats make hierarchical use of cues?

In this field experiment, Bechstein’s bats used multiple cognitive skills, each related to a specific cue type (object-specific, spatial and social). We provide evidence that the relative importance of each cognitive skill differed according to the circumstance, as shown for other animal species (Herborn et al. 2010; Morawetz et al. 2013). How animals prioritize one cognitive skill over another depends on the relevance of the cue and can be explained by the ‘rule-of-thumb’ approach (Fawcett and Johnstone 2003; Morawetz et al. 2013). Rules-of-thumb provide decision strategies based on different cues ranked according to their relevance in a given circumstance, thus favoring a particular cognitive skill. In our experiment, the echo-acoustic characteristics of the boxes were perceptually relevant available cues for the discrimination of roost types, favoring associative learning in the case of novel roosts. However, for re-localizing known roosts, bats seemed to prioritize spatial memory over associative learning. This is intuitive as spatial cues would be more reliable for re-localizing roosts given the spatio-temporal stability of the roosts (Lučan et al. 2009). A hierarchical use of spatial memory over associative learning has also been observed in the foraging behavior of nectar-feeding animals such as carpenter bees (Orth and Waddington 1997), hummingbirds (Hurly and Healy 1996) and bats (Thiele and Winter 2005; Stich and Winter 2006; Carter et al. 2010) whose foraging resources are also relatively predictable in space and time. On the contrary, animals preying on unpredictable resources (e.g., swarms of insects) rely more on associative learning than on spatial memory to localize known resources (Hulgard and Ratcliffe 2014).

While the importance of associative learning of object-related cues and spatial memory depended on the context, when using social information, bats improved the localization of unfamiliar roosts. In our experiment, the presence of colony members at the experimental box might be the most relevant cue. Since bats benefit from the presence of conspecifics in communal roosts for social thermoregulation (Pretzlaff et al. 2010), they should be particularly motivated to approach conspecifics. Hence, for bats that roost communally, social cues are likely to be prioritized over non-social cues when bats are searching for roosts (compare Kerth and Reckardt 2003).

Our study underlines the importance of evaluating multiple sources of information under natural conditions for a better understanding of how natural selection has shaped decision rules and the cognitive skills used for localizing resources (Houston et al. 2007; Fawcett et al. 2014).

## Electronic supplementary material

Below is the link to the electronic supplementary material.

Supplementary material 1 (DOCX 10212 kb)

Supplementary material 2 (XLSX 22 kb)
