# Supplementary material for: Free-ranging bats combine three different cognitive processes for roost localization
Source: Oecologia. 2020 Mar 31;192(4):979–88. doi: 10.1007/s00442-020-04634-8 (PMC7165157; doi:10.1007/s00442-020-04634-8)
Supplement: Supplementary file 1 — Supplementary material 1 (DOCX 10212 kb) [file 442_2020_4634_MOESM1_ESM.docx]

**Supplementary material**

Free-ranging bats combine three different cognitive processes for roost localization

Jesús R. Hernández-Montero^1^, Christine Reusch^1^, Ralph Simon^2^, Caroline Regina Schöner^1^ and Gerald Kerth^1^

^1^ Zoological Institute and Museum, Applied Zoology and Nature Conservation, Greifswald University, Greifswald, Germany

^2^ Faculty of Earth and Life Sciences, Vrije Universiteit Amsterdam, Department of Ecological Science / Animal Ecology, Amsterdam, The Netherlands

Corresponding author:

Jesús R. Hernández-Montero [jesus.hdezmontero@gmail.com](mailto:jesus.hdezmontero@gmail.com)

**Supplement S1. Experimental setup**


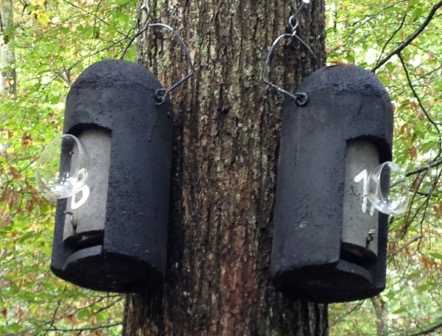


**Fig. S1.1** Experimental pair, suitable box (right) with a plastic hemisphere of 40 mm in radius; unsuitable box (left) with a plastic hemisphere of 50 mm.


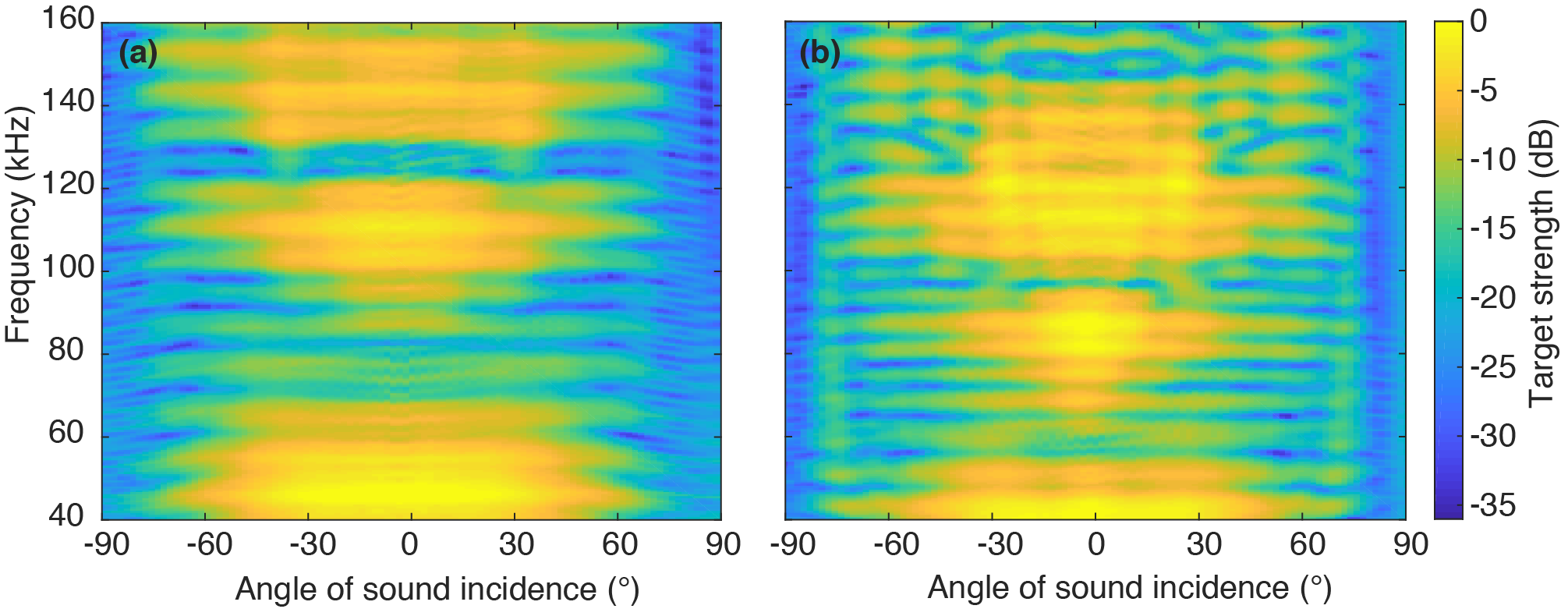


**Fig. S1.2** Echo reflectance of hemispheres in the azimuth plane. Radius a) 40 mm; b) 50 mm.

**Supplement S2. Setting a time span for categorising social information**

We assigned social information to those visits where naïve bats arrived at an experimental box together with experienced conspecifics. Later visits of experienced bats were taken into account to assess whether a naïve bat arrive within a group or alone. For defining whether a bat visited an experimental box alone or as part of a group, we calculated the time difference between two different bats arriving at experimental boxes after each other. For this purpose, we analysed the pair discovery (see manuscript for definition) events per night. Given the wide range of categories for time difference (1 – 11891 seconds), we categorised the time difference into intervals of five seconds to reduce the possible number of categories. Events above 300 seconds were assigned to an upper boundary category (>300 s) because the number of visits considerably drops after this period (Kerth and Reckardt 2003). For each box type (suitable and unsuitable), we plotted the absolute frequency and the relative frequency (percentage of cases) of every interval (Fig. S2.1), as well as the cumulative percentage of each category (Fig. S2.2). We visually explored the distribution of the data and chose the optimal time threshold to define a “social information event” by selecting the minimum possible time interval that encompasses the largest proportion of data (Fig. S2.2).

As we can see in Figure S2.1, the distribution of arrival events per time category displayed a right-skewed distribution for both types of boxes. The mean and standard deviation (mean ± SD) of the time difference between bats were 582 ± 1923 and 244 ± 1118 seconds for suitable and unsuitable boxes respectively. These values reflect the high variance in the time differences recorded. The frequency of cases for unsuitable boxes drops after 20 seconds, encompassing 83% of the data for time intervals ≤ 20 seconds. In the case of suitable boxes, the frequency of cases does not clearly drop at any specific time interval, with more than one-third of the data encompassing time intervals ≤ 30 seconds. To use the largest amount of data possible, we decided to set a one-minute as the time threshold for considering that a bat arrived together with another bat and thus social information could be used. This interval captures 88.89% and 54.25% of the arrival events at unsuitable and suitable boxes respectively (Fig. S2.2).


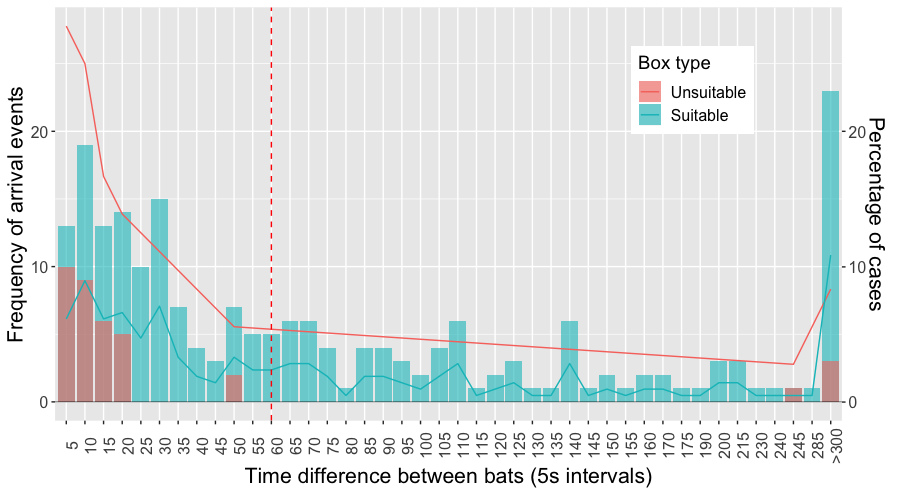


**Fig. S2.1** Frequency (bars) and relative frequency (percentage of cases, lines) of arrival events for each time interval (upper bound labelled). The vertical red dashed line signalizes 60 seconds.


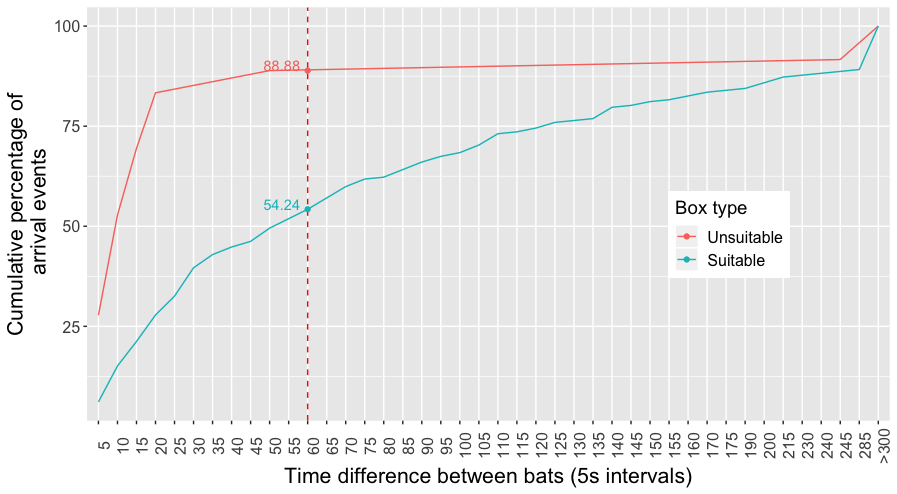


**Fig. S2.2** Cumulative percentages of arrival events per time category (upper bound labelled) for each box type. Labelled points represent the percentage of data reach within a 60-seconds span (vertical red dashed line).

**Supplement S3. Comparisons using alternative time spans**

In order to assess the robustness of our results with respect to the chosen time spans, we analysed associative learning and social information use, using alternative time spans for categorising social information. The results presented in our manuscript are based on a 60-seconds span to categorise the information type. Additionally, we computed comparisons using 30- and 180-seconds intervals.

*Associative learning*

Results on associative learning were based on the comparisons of pairs discoveries using non-social information between box types (suitable *vs* unsuitable). Bats performed more pair discoveries by visiting the suitable than the unsuitable box first (see “Non-social” column tables S3.1-3) and those differences were consistent through all analysed time spans (Fig. S3.1).


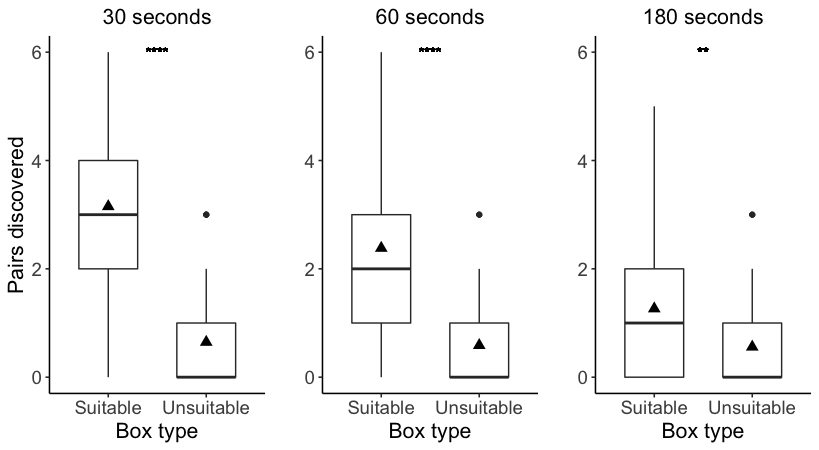


**Fig. S3.1** Comparisons using different time spans for the number of pairs discovered performed by each bat (n = 34) using non-social information. Plots are arranged column-wise under different time spans. Triangles represent the mean. Results from Wilcoxon matched-pair signed-rank test displayed as: ** *P* ≤ 0.01; **** *P* ≤ 0.0001.

*Social information use*

We assessed the influence of social information use by comparing the number of pair discoveries between information types (non-social *vs* social). These comparisons were computed for each box type.

For suitable boxes, results using a 60- and 180-seconds span were consistent. We detected a significantly higher number of pair discoveries using social than non-social information. However, we did not observe significant differences between information types using a 30-seconds span (Fig. S3.2). For unsuitable boxes, the results were consistent across all time spans, with no significant differences between information types (Fig. S3.2).

As we increased the time span used for defining information type, the number of cases using social information increased and the number of non-social cases decreased (tables S3.1-3). We decided to use a 60-second span for classifying social information events for two reasons. First, field video-records of Bechstein’s bats had shown that bats usually fly around boxes before entering them (Schöner et al. 2010). Considering this behaviour, a 30-seconds span might underestimate social events. Second, based on a previous study in Bechstein’s bats, the number of bats arriving after each other at a roost considerable decreases after 180 seconds (Kerth and Reckardt 2003). In this sense, a 60-seconds time span is more conservative for classifying social information since it is three times smaller than 180-seconds. Nevertheless, results with a 60- and a 180-seconds span were consistent.


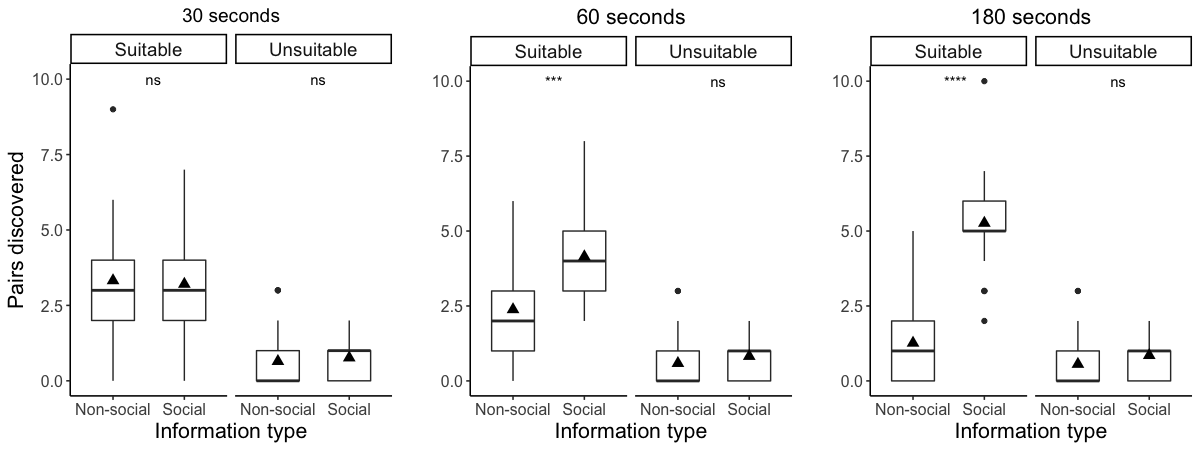


**Fig. S3.2** Box plot of the number of pairs discovered performed by Bechstein’s bats (n = 34) using non-social and social information per box type. Plots are arranged column-wise under different time spans used for categorizing social versus non-social information. Triangles represent the mean. Results from Wilcoxon matched-pair signed-rank test displayed as: ns *P* > 0.05; *** *P* ≤ 0.001; **** *P* ≤ 0.0001.

| **Table S3.1** Number of pair discoveries per box type (mean ± SD) and comparisons between information types using a 30-seconds span for categorizing information type (Z: Wilcoxon matched-pair signed-rank test; bold numbers indicate significant differences), n = 34 bats. | | | | |
| --- | --- | --- | --- | --- |
| Event | Information type | |  |  |
| Box type | Non-social | Social | Z | *P*-value |
| Pair discovery | | | | |
| Suitable | 113 (3.32 ± 1.63) | 109 (3.21 ± 1.49) | -0.207 | 0.83 |
| Unsuitable | 22 (0.65 ± 0.98) | 26 (0.76 ± 0.69) | 0.905 | 0.36 |

| **Table S3.2** Number of pair discoveries per box type (mean ± SD) and comparisons between information types using a 60-seconds span for categorizing information type (Z: Wilcoxon matched-pair signed-rank test; bold numbers indicate significant differences), n = 34 bats. | | | | |
| --- | --- | --- | --- | --- |
| Event | Information type | |  |  |
| Box type | Non-social | Social | Z | *P*-value |
| Pair discovery | | | | |
| Suitable | 81 (2.38 ± 1.44) | 141 (4.15 ± 1.48) | 3.286 | **0.001** |
| Unsuitable | 20 (0.58 ± 0.89) | 28 (0.82 ± 0.71) | 1.410 | 0.15 |

| **Table S3.3** Number of pair discoveries per box type (mean ± SD) and comparisons between information types using a 180-seconds span for categorizing information type (Z: Wilcoxon matched-pair signed-rank test; bold numbers indicate significant differences), n = 34 bats. | | | | |
| --- | --- | --- | --- | --- |
| Event | Information type | |  |  |
| Box type | Non-social | Social | Z | *P*-value |
| Pair discovery | | | | |
| Suitable | 43 (1.26 ± 1.24) | 179 (5.26 ± 1.48) | 4.949 | **< 0.0001** |
| Unsuitable | 19 (0.59 ± 0.86) | 29 (0.85 ± 0.74) | 1.745 | 0.08 |

**Supplement S4. Analysis per colony**

We surveyed two Bechstein’s bat colonies for assessing how free-ranging bats search for novel roosts. Both colonies lived in forests close to the city of Würzburg, Germany. We conducted our field experiments in two different years with the colonies BS and UA. The experiments in the BS colony took place between 29.05 to 11.09.2016 (106 days). The experiments in the UA colony took place between 14.05 to 25.08.2018 (104 days). We assessed whether both colonies showed a comparable visiting pattern to the experimental boxes placed in their respective home ranges. For each colony, we tested for differences in the number of pairs discovered per box type (suitable *vs* unsuitable) using arrivals with “non-social information” (Fig. S4.1). Data were obtained using a 60-seconds span for categorising information type.

For associative learning, in both colonies the bats showed the same behavioural differences between box types (Fig. S4.1). Given the overall similarities in the results in associative learning and social information use between colonies, we pooled the data of both colonies for further exploration.

**
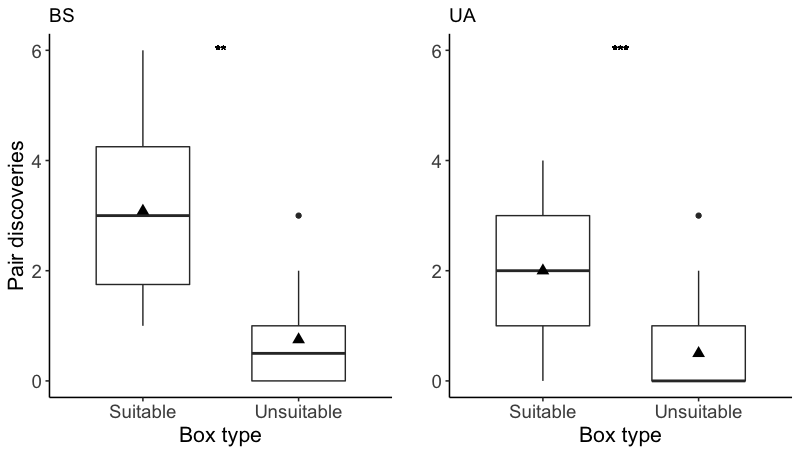
**

**Fig. S4.1** Boxplot of the number of pairs discovered per box type performed by Bechstein’s bats using non-social information in each colony (BS: n = 12; UA: n = 22). Triangles represent the mean. Results from Wilcoxon matched-pair signed-rank test displayed as: ** *P* ≤0.01; *** *P* ≤ 0.001.

**Supplement S5. General Additive Mixed Models and fit quality assessment of the top-ranked model**

| **Table S5.1** General additive mixed models ranked according to the Akaike information criterion corrected for small sample sizes (AICc), *df*: degrees of freedom; ∆AICc indicates the difference between the AICc value of each model and the top-ranked model; *w*_i_ characterizes the probability of being the best model | | | | |
| --- | --- | --- | --- | --- |
| ^a^ Model | *df* | AICc | ∆AICc | *w*_i_ |
| box type ~ s(cum_s) + s(cum_u) | 12 | 177.7 | 0.00 | 0.665 |
| ^*^box type ~ s(cum_s) + s(cum_u) + info | 13 | 179.1 | 1.37 | 0.335 |
| box type ~ s(cum_u) | 7 | 233.4 | 55.74 | 0.000 |
| box type ~ s(cum_u) + info | 8 | 235.5 | 57.78 | 0.000 |
| box type ~ s(cum_s) | 6 | 235.8 | 58.13 | 0.000 |
| box type ~ s(cum_s) + info | 7 | 236.6 | 58.89 | 0.000 |
| Intercept only | 1 | 254.7 | 77.04 | 0.000 |
| box type ~ info | 2 | 256.3 | 78.63 | 0.000 |
| ^a^ Explaining factors: cum_s: cumulative number of suitable visits, cum_u: cumulative number of unsuitable visits, col: colony (BS, UA), info: information used (social, non-social). Bat identity and colony was included as a random effect in every model.  ^*^Full model | | | | |

We assessed the fit quality of the top-ranked model using the *heat map plot* and *heat map statistic* proposed by Esarey and Pierce (2012) using the R-library heatmapFit (Esarey et al. 2016). This method enabled us to examine how closely a model’s predicted probabilities match the observed frequency of events in the data set and whether these deviations are systematic or merely noise. The heat map statistic is used to quantify the goodness of model fit by looking at the proportion of observed data points that have a one-tailed p-value less or equal to 0.1. If more than 20% of the observed data has a p-value ≤ 0.1 we can suspect that our model is misspecified (Esarey and Pierce 2012).

The heap map plot (Fig. S5.1) and the heat map statistic derived from our top-ranked model allowed us to characterise its fit quality as acceptable with 15.92% of the observations having one-tailed *P*-value ≤ 0.1. The deviations from the perfect fit (see deviations of the lines representing the modelled and the empirical data Fig. S4.1) can be attributable to the number of observations (n = 270 pair discovery events). Because we did not observe every possible combination of cumulative suitable and unsuitable visits, the observed heat map line deviates from perfect fit; however, < 20% of our observations are significantly different from the perfect fit.


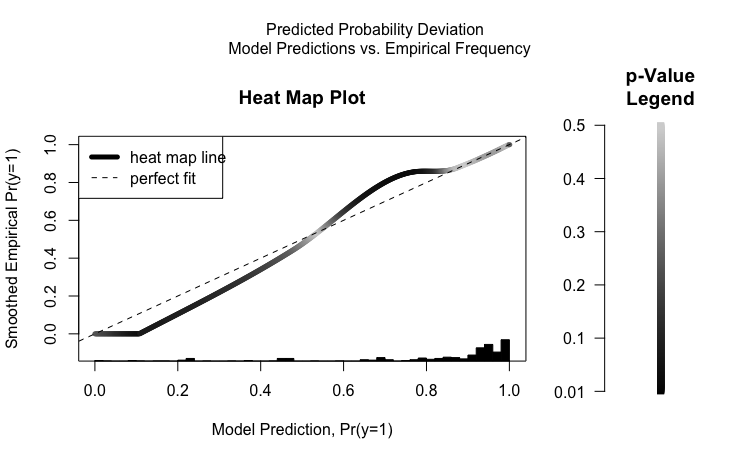


**Fig. S5.1** Heat map plot from our top-ranked model box type ~ s(cum_s) + s(cum_u), bat identity and colony were set as random factors. The histogram indicates the location and frequency of observations. Model built from 270 observations.

**Supplement references**

Esarey J, Pierce A (2012) Assessing fit quality and testing for misspecification in binary dependent variable models. Polit Anal 20:480–500

Esarey J, Pierce A, Du J (2016) heatmapFit: Fit Statistic for Binary Dependent Variable Models. R package version 2.0.4

Kerth G, Reckardt K (2003) Information transfer about roosts in female Bechstein’s bats: an experimental field study. Proc R Soc B Biol Sci 270:511–515. doi: 10.1098/rspb.2002.2267

Schöner CR, Schöner MG, Kerth G (2010) Similar is not the same: Social calls of conspecifics are more effective in attracting wild bats to day roosts than those of other bat species. Behav Ecol Sociobiol 64:2053–2063. doi: 10.1007/s00265-010-1019-8
